# Hydrogen physisorption based on the dissociative hydrogen chemisorption at the sulphur vacancy of MoS_2_ surface

**DOI:** 10.1038/s41598-017-07178-9

**Published:** 2017-08-02

**Authors:** Sang Wook Han, Gi-Beom Cha, Youngsin Park, S. C. Hong

**Affiliations:** 1 Department of Physics and EHSRC, University of Ulsan, Ulsan, 44610 Korea; 2 School of Natural Science, Ulsan National Institute of Science and Technology (UNIST), Ulsan, 44919 Korea

## Abstract

We provide a new insight that the sulphur-depleted MoS_2_ surface can store hydrogen gas at room temperature. Our findings reveal that the sulphur-vacancy defects preferentially serve as active sites for both hydrogen chemisorption and physisorption. Unexpectedly the sulphur vacancy instantly dissociates the H_2_ molecules and strongly binds the split hydrogen at the exposed Mo atoms. Thereon the additional H_2_ molecule is adsorbed with enabling more hydrogen physisorption on the top sites around the sulphur vacancy. Furthermore, the increase of the sulphur vacancy on the MoS_2_ surface further activates the dissociative hydrogen chemisorption than the H_2_ physisorption.

## Introduction

For realization of the hydrogen economy, a number of strategies have been developed to tackle key technical barriers such as hydrogen generation, storage and applications in fuel cells. Especially, the most critical problem among them is to search for suitable materials that can store hydrogen at ambient temperature and atmospheric pressure^1^. During the last few decades, numerous hydrogen storage materials were extensively investigated, including carbonaceous materials^2^, metal–organic frameworks^3^, and metal hydrides^4^. Among them, the molecular adsorption (physisorption) on the porous materials constitutes a main avenue of research due to the fast reversibility of adsorption and desorption^5^. However, high storage densities have been obtained only at temperature of 80 K or below, because of the weak van der Waals (vdW) interaction between hydrogen molecules and the surface of most porous materials^6^. Therefore, the capacity of the H_2_ physisorption at high or room temperatures is of the utmost importance.

On the other hand, the layered 2H-MoS_2_ has been intensively studied as a promising and inexpensive alternative to platinum (Pt) for the prominent hydrogen evolution reaction (HER) catalysis nearing the efficiency of Pt^7^, together with its unique electronic and optical properties^8^. It has long been realized that the HER activity stems from the coordinatively unsaturated sites at the edges of the MoS_2_, i.e., the exposed Mo sites, while the basal plane of MoS_2_ is catalytically inactive^9–11^. Hence, designing MoS_2_ nanostructures with more edge sites has become a significant topic^12, 13^. Very recently, the basal plane of monolayer 2H-MoS_2_ has been catalytically activated and optimized for the HER by applying the strain on the single sulphur vacancies (V_S_). It is understood that the dissociated hydrogen atoms or ions from the water splitting are bound at the significantly increased number of the exposed Mo atoms on the basal plane of the MoS_2_^14^. More interestingly, the first-principles calculations have predicted that the H_2_ molecule dissociates at the V_S_^15^. Furthermore, it has been reported that the hydrogen molecules adsorb dissociatively on the 4-fold symmetric Mo exposed surface of substoichiometric MoS_*x*_ phase at room temperature^16^. These results need to reveal how the V_S_ on the MoS_2_ surface intrinsically interacts with the hydrogen.

Since the predominant V_S_ defect is occasionally obtained in the MoS_2_ samples prepared by mechanical exfoliation^17^, we have investigated the hydrogen gas interaction on the various single-crystalline MoS_2_ surfaces in the UHV chamber using angle-resolved photoemission spectroscopy (ARPES) supported by density functional theory (DFT) calculations. Here we unexpectedly find that the V_S_ defects serve as active sites for both hydrogen chemisorption and physisorption at room temperature. The H_2_ molecules instantly and preferentially dissociate at the V_S_ defect on the MoS_2_ surface. Additionally more H_2_ molecules are adsorbed on the top site of the dissociative hydrogen chemisorption at the V_S_ defect with expanding the other top sites of Mo (T_Mo_), S (T_S_), and hollow, i.e., the center of a hexagon (T_H_) around the V_S_. This new insight makes attractive for onboard storage applications with providing ways to modify very small specific surface area of the MoS_2_ surface^18^ to increase both the adsorption potential and the number of available sorption sites.

**Figure 1:**
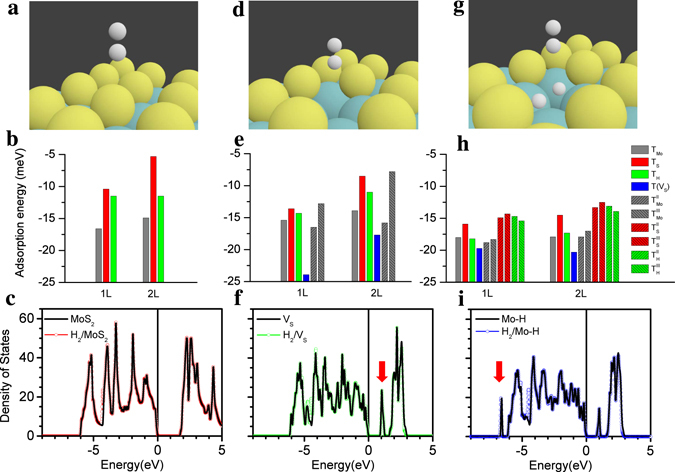
Enhancement of the hydrogen physisorption via the creation of a sulphur vacancy on the MoS_2_ basal plane. (Upper panels) Schematic of a H_2_ molecule adsorption on each surface with no defect (**a**–**c**), one S-vacancy (**d–f**) and two absorbed H atoms on the Mo atoms around the S-vacancy (**g–i**). Small (white), middle (yellow) and large (azure) balls indicate the H_2_ molecule, S and Mo atoms, respectively. (Middle panels) Comparison of the adsorption energies of H_2_ molecule on top of the Mo, S and hollow sites for mono- (left) and bilayer (right) MoS_2_ surfaces, respectively. (Bottom panels) Comparison of the total density of states with and without the H_2_ molecule adsorption. The vertical solid lines in each panel indicate the VBM being set to zero in order to clarify the bandgap.

In the defect-free MoS_2_ layers (Fig. 1a), the H_2_ molecules favor the T_Mo_ site with having an axis perpendicular to the plane^19^. However, the other positions of T_S_ and T_H_ also have very similar adsorption energies (differences < 10 meV in Fig. 1b). The equilibrium height (*h*) between the center of mass of the H_2_ molecule and the top Mo-layer of the MoS_2_ sheet is 4.65 Å. The length of bonds in the hydrogen molecule is 0.76 Å, which is a little larger than the limiting value of 0.74 Å in the ref. 20. The adsorption of H_2_ molecules on the defect-free MoS_2_ surfaces has a weak contribution at −4.3 eV of total density of states (DOSs) as shown in Fig. 1c. It is notable that the interstitial H_2_ molecules favor the T_H_ site^21^.

On the other hand, when a topmost S atom is removed, i.e., the V_S_ defect is introduced, the preferential adsorption position of H_2_ molecules changes to be the on-top site of a remained bottom S atom [T(V_S_)] with the *h*[T(Vs)] ≈ 5.20 Å [h(T_Mo_) ≈ 3.64 Å] as shown in Fig. 1d (Supplementary Fig. S1). The defect concentration is calculated using the 3 × 3 unit cell and estimated to be 5.56 and 2.78% for the mono- and bilayer systems, respectively. Interestingly, the on-top sites of second nearest Mo positions (${{\rm{T}}}_{{\rm{Mo}}}^{{\rm{II}}}$) surrounding the V_S_ have slightly lower adsorption energies than that of the first one (T_Mo_) as shown in Fig. 1e. In addition, the difference of adsorption energies between the T(V_S_) and ${{\rm{T}}}_{{\rm{Mo}}}^{{\rm{II}}}$ sites conspicuously reduces in the bilayer than in the monolayer. More interestingly, the presence of the V_S_ introduces a defect state in the bandgap as indicated by (red) arrow in Fig. 1f. This new feature originates from the excess electrons of the three unsaturated Mo atoms surrounding the V_S_. Nevertheless, the adsorption of H_2_ molecules on the defective MoS_2_ surface also has a small influence on the DOSs (Fig. 1f).

On the other hand, it has been found that the H_2_ molecules dissociate at the V_S_^15^. The dissociative hydrogen chemisorption is more stable with the significantly reduced adsorption energies of −0.441 eV (−0.444 eV) for monolayer (bilayer) system, compared to the physisorption of H_2_ molecule at the T(V_S_) site (Fig. 1e). When the H_2_ molecule is dissociated with distance of 1.67 Å (Fig. 1g), each H atom forms a bridge (Mo-H) bond between two Mo atoms (1.78 and 2.02 Å, respectively) of three Mo atoms around the V_S_ (Supplementary Fig. S2)^15^. In the DOSs, the defect states in the bandgap (Fig. 1f) are weakened and broadened (Fig. 1i) due to the compensation of the excess electrons of the three unsaturated Mo atoms surrounding the V_S_. Accordingly, the Mo-H bonds introduce additional new states at the tail of the DOSs as indicated by (red) arrow in Fig. 1i. Moreover, based on the absorbed hydrogen atoms, when the additional H_2_ molecule is located on the T(V_S_) site (Fig. 1h) with the *h*(T_S_) = 5.31 Å [*h*(T_Mo_) = 3.80 Å] (Supplementary Fig. S1), adsorption energies of the other positions are remarkably reduced compared to the previous case (Fig. 1g). This implies that the available adsorption sites of the H_2_ molecules sequentially extend to neighboring T_Mo_, T_H_ and T_S_ sites around the V_S_ defect. The H_2_ physisorption based on the dissociative chemisorption (Fig. 1g) is reversely corresponds to the mechanism or reaction pathways of HER with defective MoS_2_ catalysts (Volmer-Heyrowsky-Tafel mechanism).

**Figure 2:**
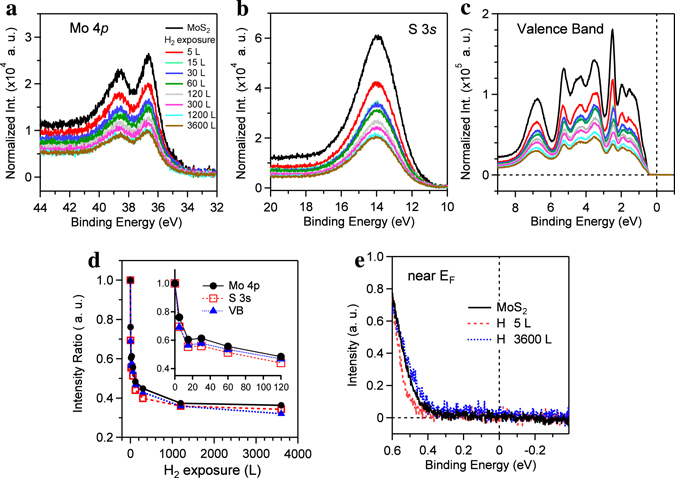
Reduction of the photoemission spectra by the hydrogen physisorption. Hydrogen gas adsorption on the cleaved MoS_2_ surface *in-situ* at room temperature. Photoemission spectra of Mo 4p (**a**) and S 3 s (**b**) shallow core level, and valence band (**c**) were obtained at *hv* = 100 eV. (**d**) Plot of the relative intensity as a function of the H_2_ exposure time. The inset shows a magnified region. (**c**) Valence-band maxima near the Fermi energy were obtained with high-resolution energy.

Figure 2a–c show the shallow core-level spectra of Mo 4p and S 3 s, and the valence-band spectra, respectively. Interestingly, without the shifts of binding energies, all intensities of the photoemission spectra for the cleaved MoS_2_ surface decreased rather quickly when exposed to the hydrogen gas in the UHV chamber at room temperature. In Fig. 2d, all intensities are halved in 60 s (60 L, 1 L = 10^−6^ Torr•s) and further reduced to 34% in 1 h (3600 L). The entire surface of the cleaved MoS_2_ is supposed to be fully and physically covered with hydrogen gas.

**Figure 3:**
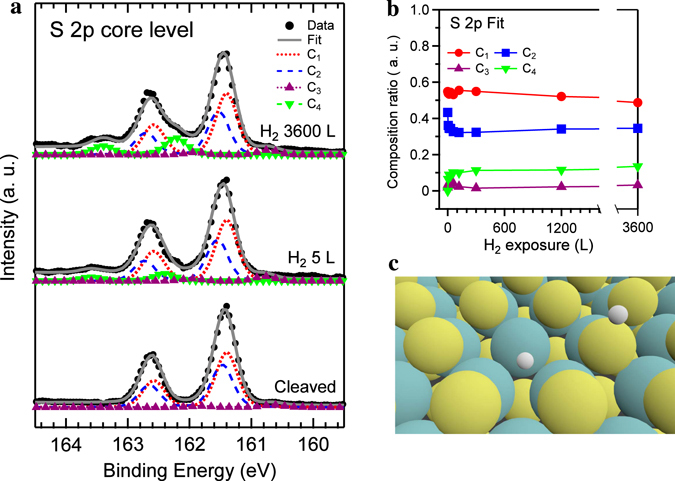
Evidence of the chemisorbed hydrogen at the S 2p core-level spectra. (**a**) S 2p core-level photoemission spectra (solid circles), obtained by the 2^nd^ light order of the photon energy of 100 eV, along with the curve-fitting results. (**b**) Plot of the relative intensity for the four components, C_1_ (red), C_2_ (blue), C_3_ (green), and C_4_ (purple) as a function of the H_2_ exposure time. (**c**) Schematic of two absorbed H atoms with Mo and S atoms around the S-vacancy.

In details (Fig. 2d), however, the initial H_2_ exposure leads to slightly shift the valence band maximum (VBM) of the cleaved surface (0.45 eV) toward high binding energy side (0.51 eV) and then the further H_2_ exposure reverses the shift of VBM toward E_F_ (0.43 eV). This subtle change is more elucidated in the S 2p core-level spectra (Fig. 3a). In the curve fitting, the low and high binding-energy components of the main peak represent the subsurface or bulk (161.40 eV, C_1_, red solid line) and the surface (161.47 eV, C_2_, blue solid line) contributions, respectively^22^. As the cleaved MoS_2_ surface is exposed to the hydrogen gas, the intensity of the surface state significantly decreased from 43 to 32% (Fig. 3b), compared to the intensity change of the bulk state (from 54 to 49%). Concurrently, when the binding energy of the bulk state is fixed, that of the surface state consistently changed to follow the shift of the VBMs (Fig. 2e) with including the other components. On the other hand, we note that a very weak component is additionally required to fit the S 2p spectrum. The third component of C_3_ (red triangle-dotted line) corresponds to the low-valence-state sulphur (S¯) due to the presence of the V_S_ defect^23^. It is away from the bulk state of C_1_ by −0.75 eV, which is comparable to the energy difference between the VBM and defect state in Fig. 1f. The defect state is located at 0.78 (1.02) eV above the VBM with the bandgap of 1.25 (1.61) eV for the bilayer (monolayer). The relative intensity of the C_3_ (Fig. 3b) rarely changed from 2% to 3%. This amount is much larger than the estimated concentration of the V_S_ in the 3 × 3 bilayer because the probing beam size of the ARPES is approximately of 2 × 1 mm^2^.

More importantly, a new component of C_4_ (green triangle-dotted line) appeared at the higher binding energy side (162.41 eV) with the ${{\rm{\Delta }}}_{SOC}$ = 1.18 eV at the initial exposure of the H_2_ molecules (5 L). The relative intensity further increased from 6 to 14% at the H_2_ exposure of 3600 L. The energy difference between the C_1_ and C_4_ components (0.7~1.0 eV) is quite comparable to that (0.6 eV) between the Mo-H bonds-induced peak (−6.6 eV) and the edge of main DOSs (Fig. 1i). Thus, this new feature is considered to be related to the dissociative chemisorption of H_2_ molecules (Fig. 1g) together with the VBM shift at the initial stage (Fig. 2e). On the other hand, this feature of the dissociative hydrogen chemisorption also appeared at the Mo 3d spectrum together with the S 2p spectrum when another sample was directly annealed at 300 °C in the H_2_ gas ambient (Supplementary Fig. S3). This implies the formation of the mixed Mo-H and S-H (Fig. 3c) bonds around the V_S_ defect. The S-H bonds on the MoS_2_ surface is an endothermic process with the adsorption energies of 0.656 eV, while it is an exothermic process (−0.12 eV) at the coordinatively unsaturated Mo edge sites^24^. On the other hand, it is notable that the hydrogenation of the MoS_2_ single crystal by the S-H bonds induces the weak ferromagnetism and formation of the atomic stripes on the MoS_2_ surface^25, 26^. The further hydrogenation at higher temperature (above 500 °C) resulted in more severe decomposition of MoS_2_ with H_2_S desorption^27^. Thus, instead of the annealing, in order to effectively desorb the physically adsorbed H_2_ molecules, the alternative method such as the application of the compressive strain is considered, because it returns to the poor activity of the basal plane^28^.

**Figure 4:**
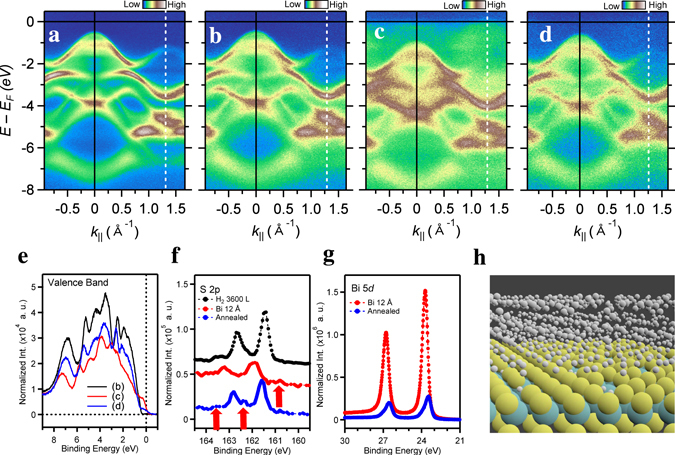
Estimation of the adsorbed hydrogen coverage on the MoS_2_ surface. (**a**)–(**d**) ARPES intensity maps were measured along the $\overline{\Gamma K}$ high-symmetry lines before (**a**) and after (**b**) the 3600 L H_2_ exposure on the cleaved MoS_2_ surface, and subsequently Bi deposited on the surface of (**c**), and then annealed surface (**d**). (**e**)–(**g**) Valence band, S 2p and Bi 5d core-level spectra. (**h**) Schematic of the monolayer of physisorbed H_2_ molecules based on chemisorbed H atoms on the monolayer MoS_2_ with S-vacancies.

On the other hand, the comparison of the ARPES data between the cleaved (Fig. 4a) and H_2_ exposed (Fig. 4b) MoS_2_ surfaces consistently shows delicate differences due to the hydrogen interaction. The overall band structure of the cleaved surface became diffuse after the H_2_ exposure of 3600 L. The dip states (−2.35 eV) around $\bar{K}$ point in Fig. 4a, corresponding to those between the third and fourth peaks in Fig. 2c, were filled and blurred in Fig. 4b. It seems to be related to the weak contribution at −4.3 eV of DOSs due to the H_2_ physisorption. Additionally, the tail of the band structures at the $\bar{\Gamma }$ point extends toward higher binding energy side. This is related to the additional new state due to the chemisorbed H atoms at the nearby Mo atoms around the V_S_ as shown in the DOSs of Fig. 1i and the C_4_ feature in Fig. 3a.

Finally, in order to confirm the hydrogen physisorption and estimate the coverage, we performed the subsequent bismuth (Bi) deposition on the hydrogen covered MoS_2_ surface. By the Bi deposition, the VBM (0.43 eV) of the 3600 L H_2_ exposed MoS_2_ surface was shifted away from E_F_ by 0.51 eV with partially occupied Bi related-bands at E_F_ (Fig. 4c), and then reversely moved toward E_F_ by −0.36 eV after annealing at 200 °C for 30 s (Fig. 4d). Accordingly, all the photoemission intensities were further reduced to 1/3 and then recovered to 2/3 of that before the Bi deposition (Fig. 4e and f). Notably, due to the photoionization cross section at the current photon energy^29^, the partially occupied, weak contribution of the Bi p orbitals near E_F_ is distinct at the valence-band spectra (Fig. 4e). In addition, the C_4_ feature of the S 2p spectrum was still retained even after annealing, together with the remained C_3_-like feature after Bi deposition (arrows in Fig. 4f). Whereas, the intensity of the Bi 5d spectrum (Fig. 4g) was reduced to 1/5 after annealing. The ratio difference of the latter is originated from the strong hybridization of the Bi clusters on the MoS_2_ surface (Details will be published elsewhere). First of all, the Bi induced electron doping into the MoS_2_ surface elucidates that the H_2_ molecules are adsorbed physically on the MoS_2_ surface. And we notice that the reduced intensities are quite similar to the case of the Bi deposition on the cleaved MoS_2_ surface, i.e., without the hydrogen exposure at the same condition of the Bi thickness (not shown here). This ensures that the desorbed H_2_ molecules are no more than the monolayer, because the probing depth at the current photon energy is comparable to the thickness of 1L-MoS_2_ (~6.5 Å) at least^30^. Based on these results, we could materialize the H_2_ molecules covered MoS_2_ surface based on the dissociative chemisorption of H atoms around the V_S_ defects in Fig. 4h. The heights of the physisorbed H_2_ molecules are 3.57 (T_H_), 7.31 (T_Mo_), and 10.34 Å (T_S_), respectively, with respect to the Mo plane. They have the tilt axes to the basal plane due to the interaction among the H_2_ molecules. The estimated coverage of the H_2_ physisorption on the 3 × 3 monolayer MoS_2_, i.e., a gravimetric storage density is of 3.6 wt.%, which is much higher than the capacity of the MoS_2_ nanotubes^31^.

For practical application, we additionally note that the presence of the carbon impurity, especially the formation of the hydrocarbon on the air-exposed surface, is a crucial parameter to prohibit the MoS_2_ surface from interacting with the hydrogen (Supplementary Fig. S4). The carbon impurity is also occasionally obtained even on the cleaved MoS_2_ surfaces with having the n-type conductivity (Supplementary Fig. S3), which is contrary to the current p-type surface. In fact, the p-type feature has been more elucidated on the more defective surface^32^. Consistently, the increased V_S_ concentration has reduced the bandgap^14, 17^ and enhanced the adsorption strength on the V_S_ sites^14^. Moreover, the additional PES measurements and DFT calculations on the more defective MoS_2_ surface (Supplementary Figs. S5 and S6) reveal that the increase of the V_S_ concentration further activates the dissociative hydrogen chemisorption than the H_2_ physisorption. These results explain the reason of the dissociative hydrogen chemisorption at the substoichiometric MoS_*x*_ surface^16^.

Our results provide a concrete possibility that the control of sulphur vacancies on the MoS_2_ surface opens up a new route for the next hydrogen storage working at room temperature and possibly more capacity at lower temperature or pressure.

## Methods

### ARPES measurements

ARPES and photoemission experiments were performed at the 4A2 and 10D beamlines of the Pohang Accelerator Laboratory (PAL), respectively. All photoemission data were collected at room temperature. The energy and angle resolutions of the ARPES apparatus (4A2) for the ARPES data obtained at the photon energy of 100 eV were better than 130 meV and 0.4°. Natural, single crystalline MoS_2_ samples (SPI) were cleaved in the UHV chamber at a base pressure better than 2 × 10^−10^ Torr and then exposed to the H_2_ gas by filling the chamber with a pressure of 1 × 10^−6^ Torr. The cleanliness and structural order were verified by the quality of the valence band dispersion and low energy electron diffraction (LEED) pattern.

The S 2p core-level photoemission spectra were obtained by the 2^nd^ light order at the photon energy of 100 eV. For comparison, they were shifted by + 101.3 eV after the energy calibration. In fitting of S 2p core-level spectra^23^, the natural (Lorentzian) line width, representing the core-hole lifetime, was determined to be ~0.07 eV while the Gaussian width was fixed at the instrumental resolution of 380 meV. The values of the spin-orbit coupling (${\rm{\Delta }}{E}_{SOC}$) and the branching ratios I(2p_3/2_)/I(2p_1/2_)] were 1.18 eV and 0.5, respectively.

### DFT calculations

In order to understand how the presence of V_S_ defects fundamentally alters the catalytic property of the basal plane of MoS_2_, we calculated the adsorption energies of the H_2_ molecules on mono- and bilayer MoS_2_ samples with and without V_S_ defects on each surface. The DFT calculations were performed by adopting the generalized gradient approximation (GGA) of the PBEsol^33^ functional for the exchange correlation potential and the projector augmented wave (PAW) method^34^ as implemented in the Vienna Ab initio Simulation Package (VASP)^35^. The electron wave function was expanded in a plane wave basis set with an energy cutoff of 400 eV. A vacuum region is thicker than 10 Å in order to avoid the coupling of the interlayer. Integration over the Brillouin zone was carried out by using 15 × 15 × 1 Monkhorst-Pack k-point mesh for all systems considered. All atomic positions for 3 × 3 cell of mono- (*a* = 3.141 Å) and bilayer (*a* = 3.142 Å) MoS_2_ were fully optimized. For comparison of the calculations with and without including the vdW interaction, the optB86b-vdW^36^ functional was also calculated with the lattices of mono- (*a* = 3.163 Å) and bilayer (*a* = 3.165 Å) MoS_2_ (Supplementary Fig. S7).

## Electronic supplementary material

Supplementary Information
