# Supplementary material for: Hydrogen physisorption based on the dissociative hydrogen chemisorption at the sulphur vacancy of MoS2 surface
Source: Sci Rep. 2017 Aug 2;7:7152. doi: 10.1038/s41598-017-07178-9 (PMC5541097; doi:10.1038/s41598-017-07178-9)
Supplement: Supplementary file 1 — Supplementary Information [file 41598_2017_7178_MOESM1_ESM.pdf]

Supplementary information on

Hydrogen physisorption based on the dissociative hydrogen chemisorption at the sulphur vacancy of MoS_2 surface

Sang Wook Han[1,*], Gi-Beom Cha[1] , Young S. Park[2], and S. C. Hong[1,*]

[1]Department of Physics and EHSRC, University of Ulsan, Ulsan 44610, Korea

[2]School of Natural Science, Ulsan National Institute of Science and Technology (UNIST), Ulsan 44919, Korea

E-mail:swhan72@ulsan.ac.kr; schong@ulsan.ac.kr

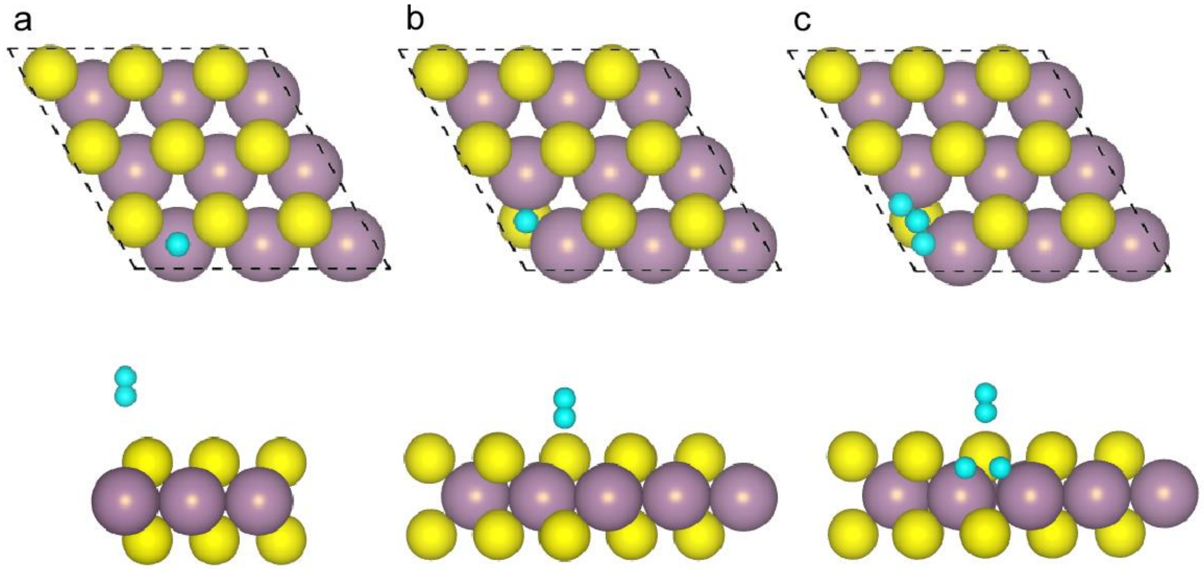

Fig. S1 Top and side views for a H_2 molecule adsorption on (a) defect-free MoS_2 surface, (b) with a single S-vacancy and (c) with two H atoms chemisorbed surface of (b). Small (white), middle (yellow) and large (azure) balls indicate the H_2 molecule, S and Mo atoms, respectively.

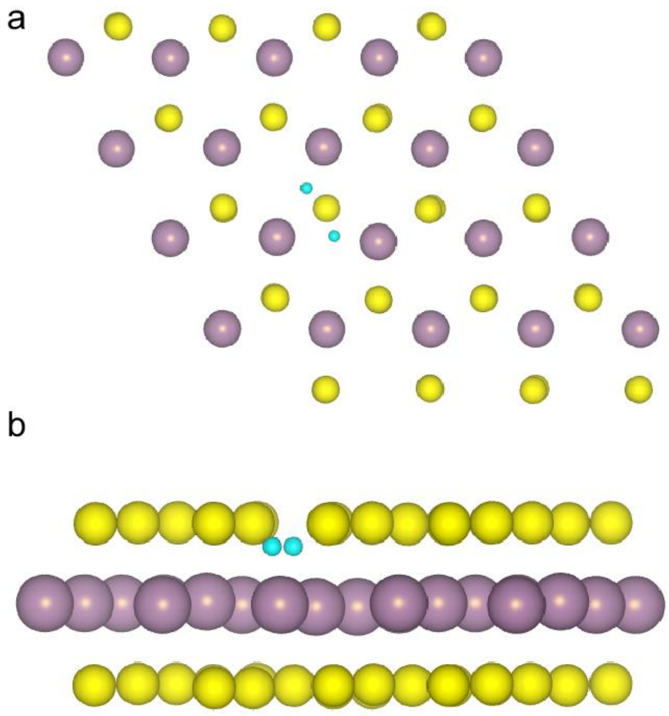

Fig. S2 (a) Top and (b) side views for two H atoms chemisorbed on the Mo atoms around a S-vacancy. Two H atoms are dissociated with distance of 1.67 Å and each H atom forms a bridge (Mo-H) bond between two Mo atoms (1.78 and 2.02 Å, respectively) of three Mo atoms around the S-vacancy.

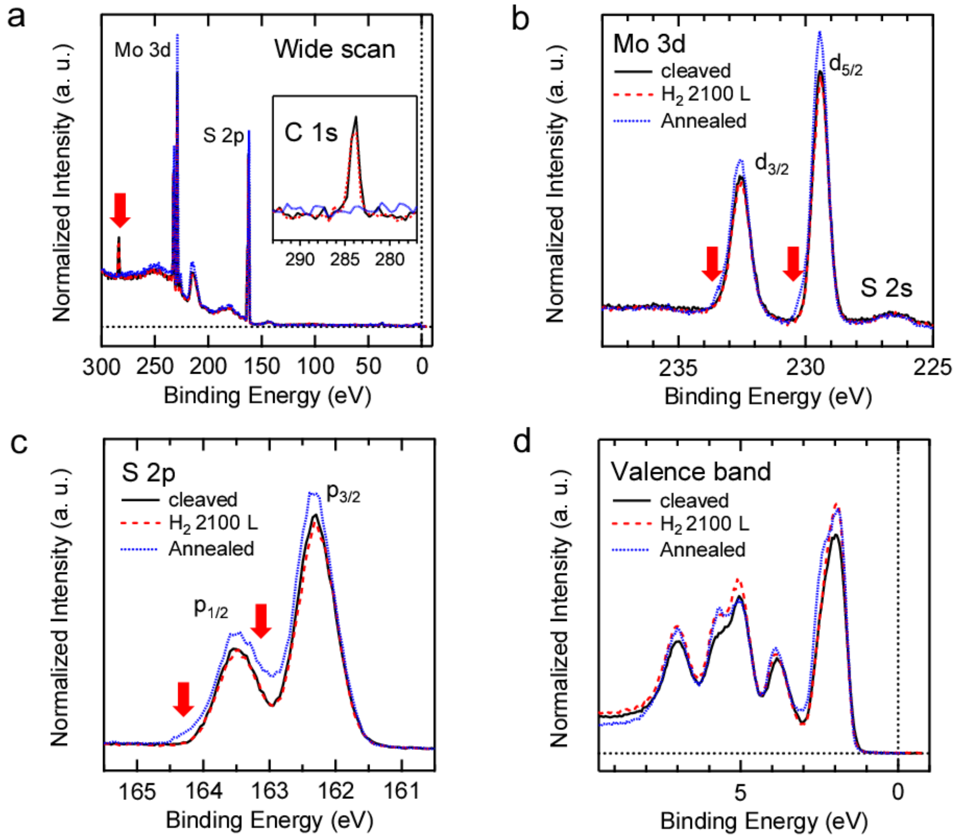

Fig. S3 (a)-(d) Photoemission spectra of wide-scan, Mo 3d and S 2p core levels, obtained at *hν* = 360 eV, and valence band, obtained at *hν* = 50 eV. The cleaved MoS_2 surface (solid lines) was exposed to the H_2 gas for 2100 L (dashed lines), and then post-annealed at 300 ^oC for 30 min., together with exposing to the H_2 gas.

Another sample was cleaved in the UHV chamber. The cleaved MoS_2 surface shows the existence of the carbon impurity in the inset of Fig. S3a. In contrast to the cleaved surface of main text, this surface was inert to the H_2 exposure of 2100 L. When the sample was directly annealed, the carbon impurity was disappeared, together with exposing to H_2 gas. After annealing[1], all the spectra were shifted toward E_F. For comparison, they were aligned to those before annealing; +0.52 eV for both Mo 3d (Fig. S3b) and S 2p (Fig. S3c) peaks, and +0.60 eV for valence band (Fig. S3d). Interestingly, the C_4-like feature of Fig. 3a is appeared at the higher binding energy side of both Mo 3d and S 2p core-level spectra (indicated by

red arrows) with the relative intensity ratio of 5 % to the main peaks. This means that the hydrogenation, i.e., the hydrogen bonding to the Mo and S atoms, is well activated at the elevated temperature. Notably, although the VBM was shifted from 1.35 eV to 0.75 eV after the H_2 annealing, it is still far away from E_F, to be remained in the n-type conductivity. This a clear difference of the current surface in comparison with that of the main text (Fig. 2e).

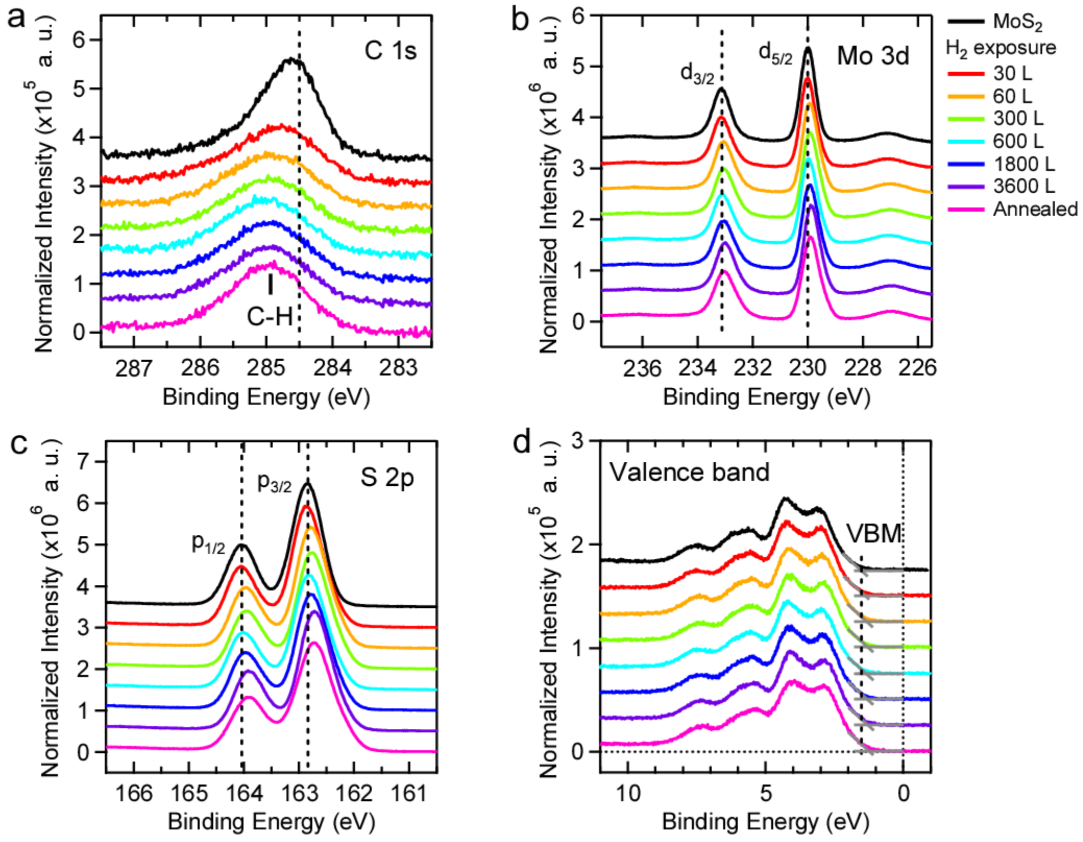

Fig. S4 (a)-(d) Photoemission spectra of C 1s, Mo 3d and S 2p core levels, and valence band, obtained at *hv* = 340 eV. The air-exposed MoS_2 surface was exposed to the H_2 gas for 3600 L, and then post-annealed at 300 °C for 1h, together with exposing to the H_2 gas.

Figure S4a shows that the binding energy of the adsorbed carbon on the air-exposed MoS_2 surface moves from 284.60 eV to 285.00 eV with a reduced intensity after the initial H_2 exposure (30 L), resulting in the reaction of hydrocarbon on the surface. And then, although the binding energies of all

spectra are slightly shifted toward E_F at the further exposure of H_2 molecules or even after the hydrogenation, the intensities are remained constant. The strong hydrocarbon seems to make the MoS_2 surface catalytically inactive.

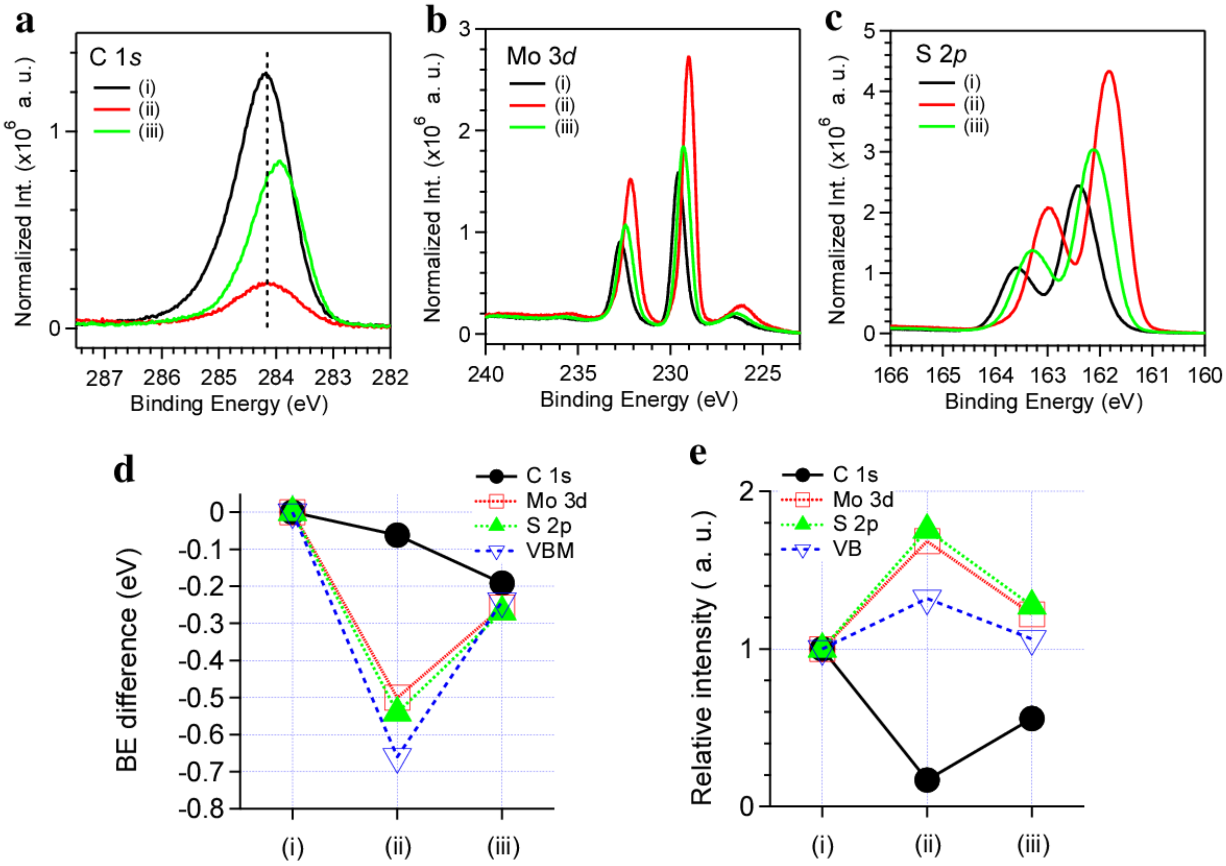

Fig. S5 (a)-(c) Comparison of the C 1s, Mo 3d and S 2p core-level photoemission spectra of the MoS_2 surface (i) as exposed to air, (ii) cleaved in the UHV chamber (2×10^{-10} Torr), and then (iii) exposed to hydrogen gas ambient (300 L). (d) Relative binding energy (BE) and (e) intensity differences. BEs of the air-exposed surface; C 1s (284.2 eV), Mo $3d_{5/2}$ (229.5 eV), and S $2p_{1/2}$ (162.4 eV) core-level peaks, and VBM (0.98 eV). They were obtained at $h\nu$ = 360 eV.

It has been reported that the mechanical exfoliation of the MoS_2 sample occasionally creates the V_S defect[2], the increase of the V_S density reduces the bandgap and enhance the adsorption strength on the

V_S sites[3]. Thus, in order to clarify the intrinsic effect of the increased V_S density on the cleaved MoS_2 surface, we repeatedly performed the PES measurements by cleaving the several MoS_2 samples in the UHV chamber. Figures S5a-c show the changes of the C 1s, Mo 3d and S 2p core-level spectra, which were obtained at the air-exposed MoS_2surface (i), cleaved surface in the UHV chamber (ii), and then exposed to the hydrogen gas (iii). All PES measurements were performed at room temperature. The influence of the hydrogen interaction on the air-exposed MoS_2 surface (i) is explained in Fig. S4. In Fig. 4d, the Mo 3d and S 2p core levels and valence-band spectra of the cleaved MoS_2 surface (ii) moved toward the high binding energy side by ~0.4 eV after exposing to the H_2 gas (iii). It is likely the electron doping. Their intensities were also decreased as shown in Fig. S5e. In addition, the full width at half maximum of both Mo 3d and S 2p was slightly increased due to the hydrogen bonds (see Fig. S3). Notably, the change of these spectra is in contrast to that of the C 1s spectrum. After exposing to the H_2 gas at this condition, the spectrum of the carbon impurity moved to the low binding energy side (Fig. S5d) with increasing intensity (Fig. S5e). Although the carbon impurity can reduce the intensities of the MoS_2 surface-related spectra, it is difficult to understand the contrasting binding energy shift of those spectra with the C 1s spectrum (see Fig. S4).

On the other hand, we note that the VBM (0.22 eV) of the cleaved surface (Fig. S6a) is very closer to the E_F than that of Fig. 2e (0.45 eV). This is consistent with that the more defective MoS_2 surface exhibits the p-type feature[4]. However, after the H_2 exposure, the VBM moved to be ~0.69 eV. This is in contrast with the H_2 physisorption showing no (binding) energy shifts in the calculations of the one V_S defect (Fig. 1) and PES spectra (Fig. 2). In order to understand these conflicting results, we performed the DFT calculations. Similar to the line defect[5], in the case of the two V_S defects on the 3×3 monolayer, the adsorption energy is dramatically reduced to -1.143 eV for the dissociative chemisorption of H_2 molecule. The bandgap of the DOSs is of 1.45 eV, which is smaller than that (1.61 eV) of the system having one Vs defect (Fig. 1f). On the other hand, when two H atoms are bonded with the exposed Mo atoms around two V_S defects, the DOSs are shifted toward low (high binding) energy side by -0.78 eV. These results elucidated that the dissociative hydrogen chemisorption is more

activated than the H_2 physisorption as the V_S density increases. These results explain the reason of the dissociative hydrogen chemisorption at the substoichiometric MoS_x surface[6].

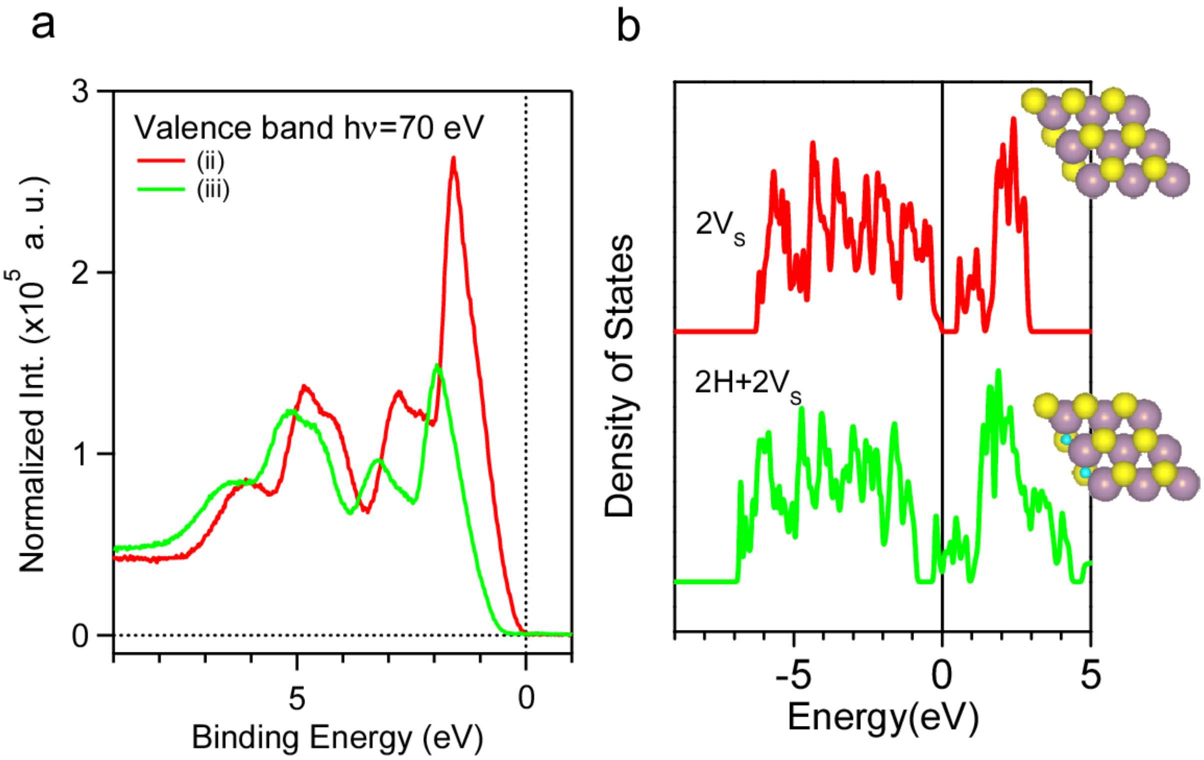

Fig. S6 (a) Comparison of the valence-band spectra of the MoS_2 surface at the stages of (ii) and (iii) as described in Fig. S5. (b) Top and bottom DOSs for two V_S defects and two H atoms chemisorbed on the exposed Mo atoms.

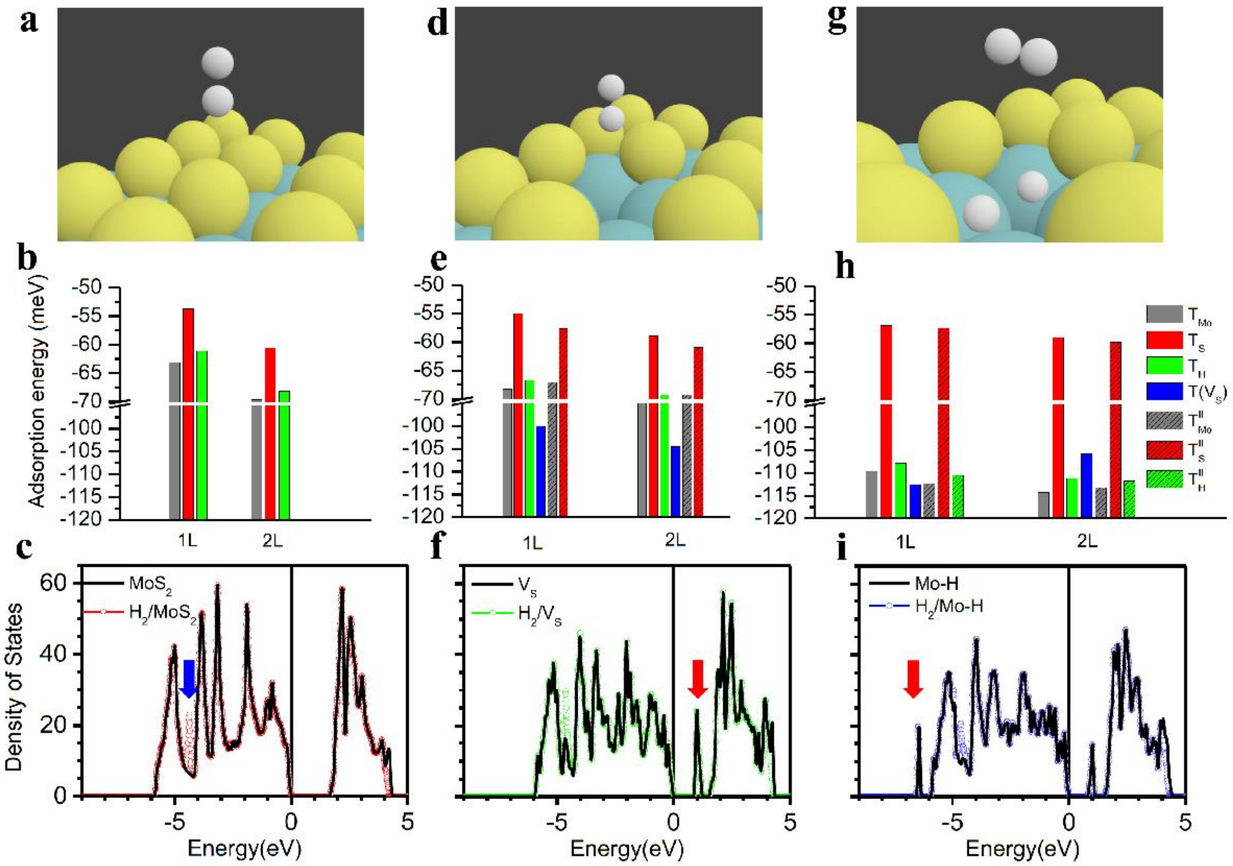

Fig. S7 Calculations by including van der Waals interaction. (a) The equilibrium height (*h*) between the center of mass of the H_2 molecule and the top Mo-layer of the MoS_2 sheet is 4.53 Å. The length of bonds in the hydrogen molecule is 0.75 Å. (d) $h[T(Vs)] \approx 5.33$ Å and $h(T_{Mo}) \approx 3.75$ Å. (g) $h(T_S) = 5.42$ Å and $h(T_{Mo}) = 3.88$ Å. The hydrogen molecule is tilted with an angle of 46.15°. (Middle panels) Comparison of the adsorption energies of H_2 molecule on top of the Mo, S and hollow sites for mono- (left) and bilayer (right) MoS_2 surfaces, respectively. (Bottom panels) Comparison of the total density of states with and without the H_2 molecule adsorption. The vertical solid lines in each panel indicate the VBM being set to zero in order to clarify the bandgap.

The DFT calculations based on the optB86b-vdW functional[7] result in much higher adsorption energies (Figs. S7) than those of the PBEsol functional (Fig. 1). For example, the adsorption energy of the dissociative hydrogen chemisorption is of -0.212 eV (-0.223 eV) for monolayer (bilayer) system (Fig.

S7g). Additionally, the dissociative chemisorption of H_2 molecule in the case of the two V_S defects on the 3 × 3 monolayer results in the adsorption energy of -0.925 eV. More remarkably, the adsorption energy of the T_S position is higher than those of the other sites and remains almost unchanged even after the creation of the V_S. Especially, the T_{Mo} and T_H sites in the bilayer system (Fig. S1h) have more reduced adsorption energies than that of the T(V_S) site. These results including vdW interaction imply that the Ts site is unfavorable for the H_2 physisorption. However, it is in contrast with the PES results showing the reduction of the S 3s and 2p core-level spectra (Figs. 2 and 3). This difference is supposed to be related to the increased lattices in the vdW calculations.

References

1. Han, S. W., Yun, W. S., Lee, J. D., Hwang, Y. H., Baik, J., Shin, H. J., Lee, W. G., Park, Y. S. & Kim, K. S. Hydrogenation-induced atomic stripes on the 2H-MoS_2 surface. *Phys. Rev. B* **92**, 241303(R) (2015).

2. Hong, J., Hu, Z., Probert, M., Li, K., Lv, D., Yang, X., Gu, L., Mao, N., Feng, Q., Xie, L., Zhang, J., Wu, D., Zhang, Z., Jin, C., Ji, W., Zhang, X., Yua, J. & Zhang, Z. Exploring atomic defects in molybdenum disulphide monolayers. *Nature Commun*. **6**, 6293 (2015).

3. Li, H., Tsai, C., Koh, A. L., Cai, L., Contryman, A. W., Fragapane, A. H., Zhao, J., Han, H. S., Manoharan, H. C., Abild-Pedersen, F., Nørskov, J. K. & Zheng, X. Activating and optimizing MoS_2 basal planes for hydrogen evolution through the formation of strained Sulphur vacancies. *Nature Mater*. **15**, 48–53 (2016).

4. Addou, R., McDonnell, S., Barrera, D., Guo, Z., Azcatl, A., Wang, J., Zhu, H., Hinkle, C. L., Quevedo-Lopez, M., ALshareef, H. N., Colombo, L., Hsu, J. W. P. & Wallace, R. M. Impurities and electronic property variations of natural MoS_2 crystal surfaces. *ACS Nano* **9**, 9124–9133 (2015).

5. Le, D., Rawal, T. B. & Rahman, S. Single-layer MoS_2 with sulfur vacancies: Structure and catalytic application. *J. Phys. Chem. C* **118**, 5346—5351 (2014).

6. Bao, Y., Yang, M., Tan, S. J. R., Liu, Y. P., Xu, H., Liu, W., Nai, C. T., Feng, Y. P., Lu, J. & Loh, K. P. Substoichiometric molybdenum sulfide phases with catalytically active basal planes. *J. Am. Chem. Soc.* **138**, 14121−14128 (2016).

7. Klimeš, J., Bowler, D. R. & Mechaelides, A. Van der Waals density functionals applied to solids. *Phys. Rev. B* **83**, 195131 (2011).
